# Questioning the ‘freedom of contract’: contract disobedience and the tenant struggle in Barcelona

**DOI:** 10.1080/27706869.2024.2310363

**Published:** 2024-02-01

**Authors:** Marta Ill-Raga

**Affiliations:** Department of Conflict and Development Studies, University of Ghent, Ghent, Belgium

**Keywords:** Contract, property, rent theory, critical legal approaches, rent control

## Abstract

In the past decade, rental housing has emerged as a new field of financialization, highlighting the importance of rent to understanding contemporary urban dynamics. Despite rental markets expressing juridical relations, empirical research on the articulations between law and rent is absent in the existing literature. In light of critical legal approaches, the paper addresses this gap through an ethnographic exploration of conflictual residential rent relations in Barcelona. The paper argues that contracts are not only legal objects crucial for the existence and articulation of rent, but their constant opening and closing, enabled by property law, result in both judicial and invisible evictions, which increase tenant turnover, thus securing incremental rental-based streams. The contract disobedience strategy enacted by the tenant movement in Barcelona has exposed how contracts are tools of domination and poses a critique of the political and legal doctrine of the ‘freedom of contract’. This mobilisation has simultaneously articulated a political demand for implementing rent controls, which are placed in a broader, gradually emerging legal countermovement to protect habitation that finds its epitome in parliamentary debates and legislative processes.

## Introduction

“The Urban Rental Law says that rent is to be freely agreed upon between the two contracting parties, but the truth is that we are not free.” A tenant pronounced these words during the weekly tenant assembly of the Barcelona Tenant’s Union (BTU) in February 2020 The sentence was a response to another tenant facing a rent increase, who wanted to know her rights in front of this situation Her contract was about to expire, and the landlord was imposing a new contract that was 120€more expensive than the one she had Tenants fear having to move out with the consequence of losing their homes immediately after the contract expires, even more so with the added difficulties of finding an affordable place to move into in the context of rising prices and increasing overburden rates in the Barcelona metropolitan area.^1^ But tenants are particularly afraid of landlords undertaking legal action, filing for eviction, and facing a court proceeding. According to the union members, although the rent increase was considered unfair, it was legal; the only possible way to avoid the rent increase was the collective organisation of tenants, of which the empirical section of this article will provide an example.

The above is a recurrent quotidian scene during tenant assemblies in Barcelona. Tenants’ unions were created in Spain in 2017 to protect a growing tenant population navigating the waters of a roaring private rental market and mobilise tenants against abusive rental conditions (Martínez and Gil 2022; Palomera 2018). The current article sheds light on how law and, in particular, contracts act as legal objects that make rent emergent as a field of exploitation by facilitating the functioning of housing as an asset from which value is extracted. The study is the result of an ethnographic exploration of residential rent relations. Fieldwork was conducted from November 2019 until September 2021 through participant observation at the BTU. By participating at the BTU as an activist, I could attend weekly tenant assemblies, where tenants suffering from rent hikes, evictions, and other abuse receive collective advice and find ways to improve their situation through mutual aid strategies. Contract terminations or contract renewals with a rent increase, resulting in potential evictions, are the most common problems tenants face. During the ethnography, I helped tenants^2^ negotiate contractual conditions with landlords and mobilise in the face of eviction. Fieldwork revealed that contracts are key mechanisms for tenant exploitation, a mode through which landlords exert power by forcing tenant competition and circulation and increasing the potential rents extracted.

The article begins with a conceptual framework that, drawing from a Marxist understanding of the role of law in market societies, places the importance of contracts in the making and articulation of market society. In light of this approach, contracts are not neutral objects; they are the hinge that allows the fiction of the exchange between equals. The article uses this conceptual scaffolding to underscore contracts as key objects to exert power and organise rent relations.

Secondly, it offers a brief historical overview of contracts and the ‘freedom of contract’ doctrine in the residential rental market in Spain. Thirdly, different cases of tenant experiences of the contractual process are presented and examined by unfolding the analysis from the most extreme experience of the eviction event to the more complex processes of contract negotiation and disobedience. The ethnographic account of the Azora tenants will elucidate how power relations and domination are present in rental contractual relations. The last empirical section introduces the reverberations of the contract disobedience strategy in broader political-legal debates to introduce housing rights legislation that limits the freedom of contract, which constitutes a legal countermovement met with stark opposition from the real estate lobby.

## The law in rent: the conceptual relevance of contracts

This section sets out to make a theoretical link between rent, law, and contractual relations. Why is it important to consider the law when researching residential rent relations? Furthermore, what is the specific role of contracts? This section seeks to answer these questions by drawing the conceptual contours of the centrality of contracts when it comes to the articulation of rent relations. First, it defines rent and exposes the way rent theory has left largely unaddressed the role that law, and contracts in particular, play in the articulation of rent. Then, it conceptualises contracts from a critical legal and Marxist perspective and presents a theoretical perspective for the understanding of contracts as mechanisms to articulate power and maintain relations of subordination.

### Defining rent

Rent is defined as “a payment made to landlords for the right to use land and its appurtenances (the resources embedded within it, the buildings placed upon it and so on)” (Harvey 1982, 330). Rent is not the simple economic value of the direct individual relationship between an object (land) and its owner. Instead, it results from a social relationship between tenants and landlords (Haila 2016, 58), including other actors such as real estate managers or state institutions. In other words, housing, as well as the rent it contains, is a social relationship and not a thing; it is the result of the existence of the institution of private property in land (Aalbers and Christophers 2014).^3^ Following this, rent can be understood “as a dominant expression of the particular way in which societies organise people’s relationship to the land and – through the land – to one another under Western capitalism” (Christophers 2020). Let us consider the evolution of contemporary capitalism. We observe that land, and housing in particular, is being increasingly treated as a financial asset, which underpins a new logic of inequality and exploitation between the asset-haves and the asset-have-nots (Aalbers 2017; Adkins, Konings, and Cooper 2020; Haila 1988; Soederberg 2018; Wigger 2021).

Legal coding, particularly the codification of property rights, is essential to articulating the actual economic functioning of assets (Pistor 2019). Housing, and the rent derived from it, should be no exception. Nevertheless, rent theory has tended to leave aside the question of the role of law and, in particular, contracts in the existence of land rent.^4^ Instead, in a non-systematic way, rent theory has pointed to two ways in which law, and in particular, property rights, are essential to the existence of rent. First, the institution and articulation of property rights are essential, foundational ‘moments’ that open the door to future rent extraction (Andreucci et al. 2017). Secondly, institutional property regimes organise and discipline subjects and provide and transmit the ideological basis (the justification story) for the appropriation of rents (Arboleda and Purcell 2021, 5; Haila 2016). Following the latter, the article contributes to understanding the ideology of freedom of contract and contracts as legal objects crucial to the circulation of rent, as they are the hinge bringing together landlords and tenants. Among the modules that the law has traditionally used to codify nature to transform it into an asset (Pistor 2019), we argue that contracts are central to understanding rent dynamics. The article seeks to advance a perspective on the concept of contract that is useful for studying the marketization of housing.

### Conceptualising contracts

Contract conceptualization has taken three different theoretical perspectives: the dogmatic, ‘free will’ legal contract theory; the economic, transaction cost theory; and the sociological relational theory of contracts (Stapper 2021). Rent theory is at odds with the social ontology of the transaction cost theory of contracts. Rent, according to this view, would be the result of individuals signing contracts in competitive markets (Haila 2016). The methodological individualism of the economic, utility-maximising vision of contracts—and by extension, the legal doctrine that underpins it—contradicts the social-relational nature of rent defended by rent theory (ibid.). The relational theory of contracts, which understands that contractual relations are embedded in social relations, posits a similar criticism to both the doctrinal and economic approaches to contracts (Campbell 2004). The main criticism of relational contract theory is that contracts, in practice, do not take place as the doctrinal and economic approaches suggest. Ian MacNeil, its most prominent exponent, defended that “maintaining the existence of freedom of contract in an age of forms and concentration of economic and social power” was the most pressing problem of standard (legal and economic) contract theory (Campbell 2004, 3). Similar to this claim, in this article, we propose that the social life of contracts needs to be analysed.

Nevertheless, in contrast with relational contract theory, this article aims to develop something other than an alternative theory of contracts capable of explaining contractual diversity and orienting contractual practice. Taking inspiration from the Marxist legal scholarship of Pashukanis (1929), the article proposes to analyse the object of the (freedom of) contract as an abstraction that, by being imposed, has real-life effects, thus shaping social relations. In other words, it is not just that social reality does not adjust to the rationality of legal doctrine, but rather that abstract rationality, in practice, shapes social relations, which, as we will show, are not free from conflict.

We argue that the critical legal perspective, in particular insights derived from the Marxist legal theory, is helpful to illuminate the contractual logic behind the creation and extraction of rent in at least three ways: first, the historical necessity of law to underpin the market economy; second, the ideological role of law, which conceals the actual power-laden practice of legal property relations; and last but not least, the organisational power of law to determine the actual shape of social relations under a capitalist society, which goes beyond both ideology and the state power and its monopoly of violence. This definition of law does not close space for contestation; on the contrary, law, as a social practice, is imbued with political struggles that transform the contents and interpretation of the legal texts.

### The power of actually existing contracts

Critical legal studies, particularly Marxist approaches to law, argue that legal form is necessary for the social existence of market relations and commodities. Pashukanis (1929) commodity-form theory of law understands that the commodity and value forms are necessarily connected to the legal form; in other words, social (re)productive relations under capitalist market societies are organised by juridical means. A critique of the forms of accumulation, dispossession, and inequality resulting from generalised market relations and the economic categories (such as value, wage, profit, but also rent) should be espoused to a critique of the legal scaffolding that allows for its existence. A theoretical consequence is that law, or the legal form, is not a historical, eternal category, consubstantial to human nature, as neo-Kantian idealist jurists of natural law propose. It is a historically specific product of the modern advent of market societies. The centrality of law has not faded away but has become ever more present in the contemporary, neoliberal form of capitalism. Proof of that is the increasing judicialization of politics; with the legal mechanisms becoming central to exerting governance and power (see Comaroff and Comaroff 2009). As we shall later see, proof of that fact is the juridical-legal expression of conflicts around residential rent relations in Barcelona.

Moving back to the role of contracts, these are vital pieces to understanding “actually existing law,” which is the object of critical Marxist theory (Miéville 2005, 85). Contracts are essential to the functioning of the market as the “sphere of circulation.” Moreover, more broadly, contracts are central to juridical and social-productive relations: legal subjects first appear as property owners with confronted interests whose autonomous wills meet halfway through the contract (Pashukanis 1929, 121). In a capitalist market society, law and contract are crucial both for the circulation of commodities and value production—workers appear as free property owners of their labour power, which they sell to the capitalist through the labour contract. A similar situation occurs between landlord and tenants in the rental contract: tenants sell their capacity of payment—that is, the fruits of their future labour—in exchange for the use of housing owned by the landlord. The contractual exchange, from a legal perspective, presents both sellers and buyers “as free persons, equal before the law,” whose relations take place in the “very Eden of the innate rights of man” (Marx 1976, 1:280).

Behind this “mantle of equality,” contracts hide the exploitation in the production process and, as feminist scholars later added, the oppressions in the reproductive sphere where housing is placed (Bhattacharya 2017, 148). By “shaking our faith on the fundamental props of modern society” (ibid.), which are our juridical rights and their basic legal categories, such as free will, contract, legal equivalence, etc., a Marxist analysis of law is not only to uncover what the legal form conceals; that is, the fundamental inequalities behind processes of production and reproduction. Beyond ideological tools, contracts organise unequal relations by equalising the exchanged properties. In contracts such as the labour contract or the traditional marriage contract—or, as argued here, the rental contract—part of the exchanged property is “property in the person”, which implies that one person will give up herself (or her body), therefore resulting in civil subordination (Pateman and Mills 2007, 209–13). Assuming autonomy of the wills in this ‘exchange’ is misplaced, as the history of the formation of capitalist market societies accounts, enclosures—the dispossession from means of reproduction (such as common lands and resources)—and other forms of discipline forced a growing working class to enter into contractual labour relations (Perelman 2000). Therefore, juridical rights and legal categories are not just masking the reality of exploitation. They are no less real social relations of a material nature than ideological instruments (Pashukanis 1929). Contractual relations and the form of the law determine how market relations take shape. Hence, in analysing existing contractual relations, it is essential to pay attention to social relations, which may not be visible at first sight.

### Contractual freedom or subordination?

Critical legal theory sets out to make visible what the legal form renders invisible. Departing from a criticism of the legal formalist idea of the ‘free subject,’ it “draws our gaze towards the mechanisms” that articulate legal relations, the historical production of juridical relations, and their social significance (Bhandar 2015, 264). The historical emergence of freedom of contract is crucial to correctly understanding its current form and the mechanisms introduced later in the analysis. Moreover, critical legal analyses are concerned with deciphering the workings of private property under capitalist market relations. From a commodity-form perspective, the essence of private property is not outright possession of the thing owned (and exclusion of anyone else from its use), but that the property owner can alienate—that is, sell, rent or undertake—any other market transaction with his property. “Capitalist landed property, for example, does not presuppose any kind of organic bond between the land and its owner. On the contrary, it is only conceivable when land changes hands with complete freedom” (Pashukanis 1929, 127). Contracts serve this purpose. They are legal objects and, as we will show, social practices that, by making exchange possible, allow property to work in its capitalist way.

Of course, contracts, in order to have binding effects, need to be legally sanctioned, that is, embedded in legal codes and protected by state authority. Nevertheless, it is essential to note that juridical relations are distinct from direct relations of political domination, as we would find under feudal systems. The state’s coercive capacity is only the guarantee and safeguard of the legally sanctioned rights and obligations that generally respond to the impersonal power of the legal system itself (Pashukanis 1929). Therefore, social and market relations, even those of unequal nature, such as rent relations, are not the result of direct imposition by violent means of rights and obligations. The contractual logic has its own power to make most of the relationships function with no force directly involved.

Nevertheless, property, far from being atemporal, is an unstable, contested category (Bhandar 2016). The contractual process, as the case study shows, is highly contested. The nature of the contract emerges through contested property relations. Despite being a minority practice, conflict and contestation over rental contracts appear as a window into the politics of property (Cockbrun et al. 2019). Analysing the social practice of contracting rental housing through the lens of the tenant-landlord conflict can illuminate and provide a clearer understanding of how power and contestation take place in the private rental sector.

## Freedom of contract in land rent: a brief historical perspective of the Spanish case

Contracts have played a crucial role in the marketization of land. In the transition from feudalism to rural capitalism in England, a key aspect was the imposition of a system of “competitive rents” in which, wherever possible, landlords would lease land to the “highest bidder” (Wood 2002, 101). In rural Lower Burma under colonial rule, in a similar process of integration into the international market, the control of land became a fundamental basis for power, with the imposition of “strict [rental] contracts” protected by the state on farmer tenants without considering the economic hardships of bad seasons (Scott 1976, 71–72). In short, the imposition of rental contracts has historically been a critical mediating legal mechanism between landlords and tenants, a crucial element for transforming property relations in the transition to a market society.

The treatment of urban land in Spain also reflects a similar historical process. The doctrine of freedom of contract found its first legal expression in the short-lived liberal constitution of 1812. The aim was to unleash the land and its productive forces from its feudal fetters—from customs or previous norms or decrees which conditioned its use and exchange. The rule was consolidated under the 9th April 1848 Act, known as the *Ley de Inquilinatos* (Tenancy Act), which stipulated that housing and urban property owners could rent out their buildings by freely agreeing on the conditions of the tenancy with the tenant, accepting the autonomy of the will of the two contracting parties as the regulating basis (Comelles 2017). In practice, though, “the aim was to finish with the costume of not being able to evict a tenant to let the property to any other person,” allowing maximum exploitation of the housing unit (ibid., 11). The general rule of freedom of contract, that crystallised in the Civil Code of 1898, is still in effect today, making it the legal normal state of affairs.

Despite the freedom of contract being the new legal basis for economic exchange, the rule has often been amended *ad hoc* to prevent its adverse effects. After the imposition of freedom of contract in 1848, urban rent prices experienced a sharp increase (Cotorruelo 1960). They found opposition from both industrialists, for whom rents were a drag on profits, and from working-class tenants, who started organising rent strikes particularly persistently at the outset of the twentieth century, with documented examples in Bilbao (1905) (Martín 2014), Seville (Ballina 2020) and Barcelona (Ealham 2005; Rider 1986). The increasing wave of protest and social discontent led to the partial elimination of freedom of contract in urban land rent by the Bugallal Decree of 1920, which imposed a temporary rule of rent controls. Its implementation was extended during the dictatorship of Primo de Rivera in 1923 due to the popular pressure to maintain rent controls (Cotorruelo 1960). Rent control during inflationary periods and indefinite leasing finally took hold after the Civil War in the Francoist Urban Rental Law (*Ley de Arrendamientos Urbanos* [LAU]) of 1946 and the subsequent amendment of 1964, which set a permanent limit to updating rents. Freedom of contract in urban rentals was suspended until the Boyer Decree of 1985, the inaugural rule of neoliberal policies, which marked the start of the housing construction boom in Spain (López and Emmanuel 2010, 227).

Nevertheless, the context between the 1960s and early 1990s had radically changed. The Francoist project of transforming Spain from a country of proletarians into a country of homeowners had its results. Homeownership was majoritarian, representing more than three-quarters of the population in 1990. Whereas freeing contractual rules by 1985 was supposed to attract investment in a deteriorating rental market, in practice, it meant a reduction in tenant rights, which made rent even more unattractive to tenants in the context of increasing institutional support for home purchases *via* mortgage credit. This lack of protection was only slightly reverted by the 1995 amendment of the LAU, which reinstituted a minimum duration of rental contracts (five years). Nevertheless, this amendment did not change the dwindling of the tenant population, which by 2007 reached its historical low at 10.4% of the total population.

The number of tenants only started to increase after the 2008 global financial crisis (GFC), which caused the collapse of the Spanish mortgage market. The immediate result was the massive wave of foreclosures that left the poorest mortgage debtors out of the ownership threshold, expelling them to the rental market. Nevertheless, the change in trend did not only result from a change in housing demography as the passive impact of the banking system crisis but also the outcome of the so-called post-crisis resolution regime: a myriad of national-level regulations that sought to transform distressed assets such as foreclosed housing into rental housing assets ripe for investment, creating a buoyant private rental market that soon became a premium investment with high returns for finance capital (García-Lamarca 2021; Gil García and Martínez López 2021).

The resolution regime in the real estate sector, beyond the bailout of the banks, first consisted of the creation of the Spanish real estate investment trusts (REITs) in 2009, which three years later was afforded a special, tax-free regime, which had the effect of expanding the REITs market from 2012 onwards with the penetration of big corporate landlords in Spain (Guzmán 2023). Regarding tenants’ rights, the growth of the market and the demand for rental units did not translate into an enhancement of tenants’ rights. Only one year after the approval of the tax-free regime for REITs, another amendment of the LAU took place in 2013. The reform, among other things, erased tenants’ right of first refusal to facilitate the buying and selling of entire housing block portfolios, erased the control of rent increases within the duration of rental agreements, and shortened the minimum duration of rental contracts to facilitate tenant turnover (Gil García and Martínez López 2021, 8). This LAU reform was a regulated deregulation that lifted several tenant protections and left an increasing number of elements (such as the internal rent increases or the longer duration of leases) to be ‘freely’ agreed upon by the contracting parties. A fully-fledged freedom of contract doctrine was being reinstituted and was only partially reverted in a 2019 LAU amendment due to the tenant movement and the oppositional politics of the BTU. Securing tenant protections by restricting the freedom of contract was necessary to prevent tenant evictions. After an unprecedented wave of mortgage evictions due to the 2008 GFC, in 2018, evictions due to rent arrears represented 65.5% of total evictions, marking a visible shift in the housing crisis from the mortgage to the rental housing sector (CGPJ (General Council of the Judiciary) 2018).

## Tenants’ experience of the contractual process

We have just explained how lifting tenant protections and reinstituting the freedom of contract have historically been vital to inserting urban land rent and housing units in the rental market under the asset logic. More recently, the flexibilization of contractual conditions has impacted the housing crisis, which has found its expression in the increased number of evictions in the rental market.

### Backwards from the eviction event: a contract closure

One way to start analysing how the contractual mechanism has the power to hold the renting relationship together is to look at it from its most extreme expression: the eviction event. Evictions are the most visible consequence of a housing crisis. As such, they are often given priority in the academic research agenda concerned with understanding how a housing crisis unfolds: a generalised displacement of the poorest populations, their separation from their inhabiting spaces, and its devastating consequences (Desmond 2016; Rolnik 2019). Nevertheless, by focusing on eviction and its aftermath, the process of which evictions are just the most extreme event is obliterated. This section argues that evictions are the final stage of a contractual process. The rental contract, tied to the existence of private property rights on land, articulates residential rent as a form of exploitation and domination.

Evictions are legal events that result from a court decision forcing the end of a contractual relationship that brought together the holder(s)—landlords—of the landed property title and the person(s)—tenants—using it. Formally, evictions are property repossessions in which land is freed from its previous user(s) to enter a new circulation phase in the rental market. In other words, evictions are contract closures that permit the opening of new contracts so that the landed property can keep on being traded (in the form of rent) in the private rental market. Analysing an eviction event from the perspective of it being the end of a much longer process—that is, the contractual process—can illuminate the legal, bureaucratic and disciplinary state mechanisms that play into the rent relationship. These aspects of the contractual process are made evident by evictions that are met with resistance from organised tenants, bringing to the surface elements that generally bypass the eye of the witnesses and even the participants in the market game. Three aspects of evictions need to be therefore highlighted: first, evictions reveal the material conditions in which the contractual bargain takes place and the disparity of forces behind the parts; second, evictions show how the role of the police is nothing but a coercive extension of the contract discipline; and finally, most of this discipline takes place in an invisible way.

Eviction blockades are the last resort for a tenant to negotiate a new contract in front of the landlord’s antagonised position that wants to end the contractual relationship. On the day of the eviction, an assemblage of the conflicting parties comes together to evaluate the possibilities of negotiating a possible settlement. The landlords themselves are rarely present, and instead, their legal representation, together with the *comitiva judicial*—a common term for the judicial entourage composed by the court bailiffs—arrive at the place of the eviction and are met with social services and council housing workers, tenants’ legal representatives, and the tenants. Activists and organised tenants try to make their way in the negotiation, although they are often sidelined because they do not have any officially recognised role. The tenant side tries to convince the *comitiva judicial* to postpone the eviction by displaying various arguments, such as the proving of official vulnerability—a bureaucratic category defined by the income level, family structure, and other special needs of the tenant household—which would entitle tenants to special protection in such circumstances. Other arguments to postpone the eviction are the existence of alternative housing that will be available in a short time or the actual presence of a blockade, which forces the *comitiva judicial* to ask for police support, which is often unavailable in the first eviction attempt. When any of these arguments is considered valid and the eviction is ‘stopped at the door’—a common activist expression to define a successful blockade—it means that, at best, it is temporarily postponed, and the tenant has bought time to foresee a new contract, either in the same housing or in an alternative one. Eviction blockades are an emergency practice of housing unionism, aiming to level tenants’ bargaining power in front of landlords’ judicially protected right to property repossession.

**Figure 1.**
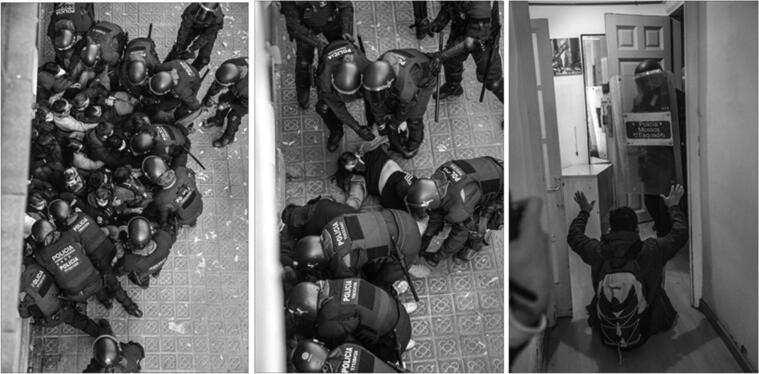
Pictures taken from carlos’s balcony inside the property being repossessed show a riot police intervention in front of an eviction blockade. Source: Ivan geisen (BTU).

When riot police intervene, blockades usually cannot stop the eviction’s enforcement. Evictions are the most coercive form of enforcement of this specific contractual relationship. The pictures above were taken during the eviction of Carlos, which took place on December 20, 2021 (see Figure 1). His rental contract had expired more than two years ago, and, since 2017, Carlos and the rest of the neighbours in his block had been organising against unilateral contract finalisations. The new property owner, a corporate landlord (Topal Trade SL), started sending the so-called *burofax*, an official notification of contract termination after which the tenants are either expected to move out or to accept new contractual conditions. In any case, the *burofax* signals the official will of the landlord to execute the end of the contract. In this case, the corporate landlord’s plan—as it happened with some of Carlos’s neighbours—was to remove the residents, renovate the flats, and offer new rental contracts after more than doubling the rent.

The police force, when it intervenes, acts as judicial police; they come to enforce the direct mandate of the court, which is the end of the rental contract. As such, police repression is a continuation of the contractual process. Nevertheless, it is essential to note that the coercive enforcement of contracts is organised not as the direct personal power of landlords but embedded in the broader legal structure that protects property rights. An anecdote during an eviction blockade on December 10, 2019 provided a clear example of the impersonality of contractual power. The *comitiva judicial* gathered at the front door of the building. The blockade that day was massive, as Livia and Juan, two tenants resisting a rent increase, had gathered much activist support. The *comitiva* finally decided to postpone the eviction blockade officially and signed the notification of suspension. Only a few seconds later, to the activists’ surprise, the police entered the building from the garage backdoor, thanks to the personal help of the landlord, who gave them access. Although the landlord tried to use police directly, the judicial officer of the *comitiva* clearly stated that the eviction had been officially and judicially postponed and sent police off. Even if “the organisation of coercion is an important element of the legal form” (Pashukanis 1929, 162), the police force is not responding to the direct mandate of the landlord; instead, the police force is organised judicially and is functional to the legal articulation of the rent relation, of which police coercive enforcement is just an extreme expression.

As tenant organisers claim, most of the contract closures, which are the same as forced displacements, go unnoticed. They are invisible evictions; for tenants, if they have an alternative, accept to move out without showing resistance or facing a no-fault eviction.^5^ Laura, the lawyer of the BTU and a public defendant, confirmed this trend during the interview as she recalled her experiences dealing with formal eviction processes. She observed that the majority of eviction cases she gets as a public defendant are rental payment defaults. No-fault evictions resulting from a contract termination are a less common cause of formal eviction.^6^ After receiving an official notification or *burofax,* tenants are normally forced to move out unwillingly. They are subject to the asset logic that is being imposed on their homes (Fields 2017), which forces an increasing tenant turnover to assure, through the opening and closing of contracts, higher returns for landlords and investors. Even if unwillingly subject to this dynamic, as we have shown, organised tenants, such as Carlos, and Livia and Juan, are showing resistance in order to subvert the contractual logic behind exploitative rent relations.

Evictions, thus, are niether market failures or expressions of extreme vulnerability nor simple, forceful impositions of property rights. Instead, evictions are necessary elements of the legal articulation of rent that are nested in a longer contractual process. As such, evictions are a mechanism to enforce tenant circulation (rather than just displacement) and, as I will present in what follows, are part of a disciplined inclusion (rather than exclusion) *via* contractual relations.

### Between contract domination and contract disobedience: Azora tenants’ case

In the previous section, we introduced evictions as the mechanism that coercively ends the contractual relationship. In this section, we are ready to explore how contracts are made and negotiated. To do so, we will illustrate the dynamics playing into the contractual process through the case of the Azora tenants and their negotiation and conflict with the corporate landlord, which took place between the autumn of 2019 and the autumn of 2020.

The first Azora tenants’ community that started organising in Badalona did so by the end of the summer of 2019, when the property informed them that the buildings had been bought by the investment fund Azora from Solvia (the previous financial landlord) under the name of its real estate company Lazora. After Azora had bought the property, it started offering new rental contracts with a rent increase of up to 80%, but through incremental rent hikes within the duration of the contract: from 1,000€to 1,300€in the first year, followed by 1,500€in the second, and finally 1,800€in the third year, thus skipping the rule that stabilises the rent during the minimal length of the rental contract, which in 2019 was extended from three to seven years.

**Figure 2.**
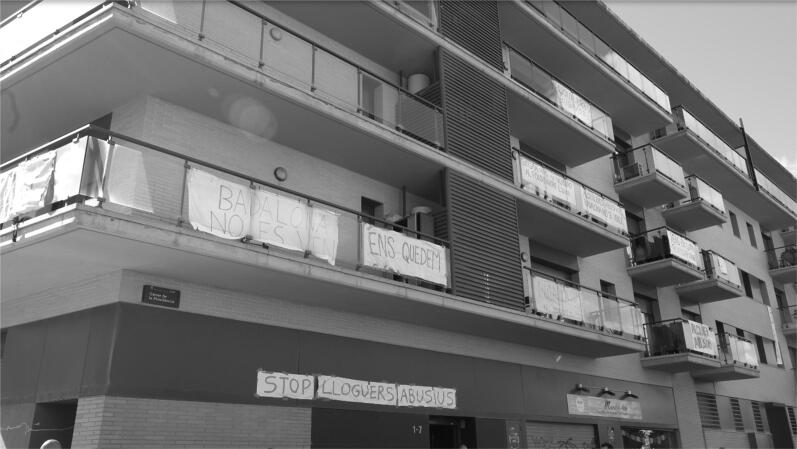
Banners hanging on the balconies of the Azora building block in Badalona. Source: author.

Shortly after the first community in the Badalona buildings started to organise, they received the support of the activists of the BTU. The first assemblies of Azora tenants started to take shape in the yard of the Badalona building, and tenants started to act. They boycotted the visits of prospective tenants to the empty building flats to let at that moment and hung banners protesting against what they framed as abusive rents. “Badalona is not for sale,” “Solvia has sold us out to the vulture fund Lazora,” and “We stay put” are among the different claims that were painted on the banners (see Figure 2).

**Figure 3.**
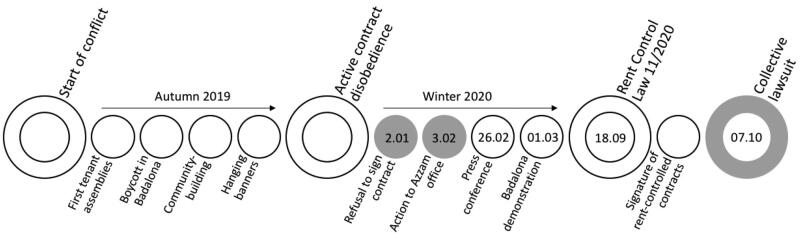
Azora tenants’ conflict chronology of key events. Source: author.

**Figure 4.**
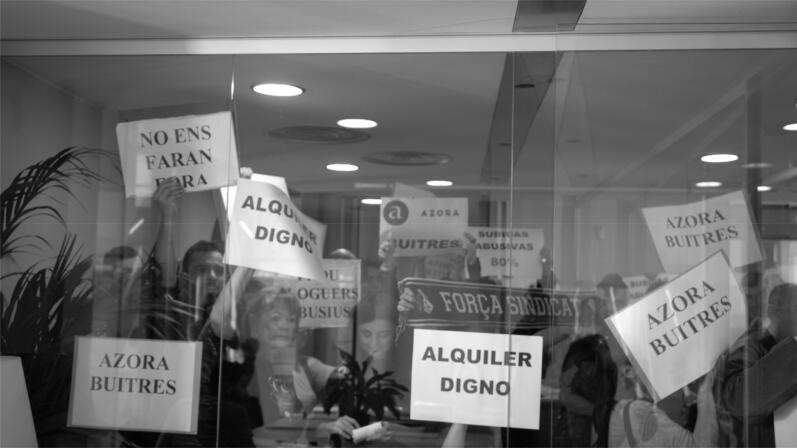
Tenants staging the protest in Azzam offices, February 3, 2020. Source: author.

As observed at the tenant assembly in Barcelona during the 22 months of fieldwork, how the Azora conflict unfolded is representative of a general, broader dynamic: rent conflicts tend to arise when there is a market transaction taking place or about to take place. Buying or selling properties or transferring property rights (such as an inheritance) are common triggers of conflicts. The expectancy of higher returns by the new landlords from their newly acquired properties activates the contractual mechanism: the imposition of a new circulation phase *via* contract closure and opening. This circulation can take mainly two forms: on the one hand, as we have noted in the previous section, it is the imposition of a contract closure that obliges the tenants to move out of their homes; on the other, the contract closure can precede a new contract offer to the same tenant with a rent increase or the inclusion of new contractual clauses adding to the instability of the tenant’s residence or reducing the tenant’s rights. The tenant problem was consequently framed around the mechanisms that permitted such a high tenant turnover: the rental contracts and the LAU. The disobedience of the two was to become the kernel of the tenants’ movement’s civil disobedience strategy, *Ens quedem* (We stay put). The main strategy steps were: firstly, when the *burofax* marking the end of the contract is received, the tenant does not obey and stays put instead of moving out or accepting a rent increase; secondly, the tenant is supposed to work towards the collectivization of the conflict, that is, looking for other tenants from the same landlord (usually in the same building) in order to create a tenant assembly; and thirdly, start a process of collective negotiation of the new contractual conditions without abusive prices or clauses.

Therefore, the general articulation of the Azora tenants’ conflict was not exceptional. What was rather exceptional was the level of collective organisation and its territorial width. Shortly after the first actions were taking place in Badalona, canvassing throughout all of the properties of Azora in the metropolitan area of Barcelona was conducted with the support of the BTU, and other buildings were organised in different localities. Properties in the cities of Terrassa, L’Hospitalet de Llobregat, Sabadell, and Granollers and the neighbourhoods of El Clot and Vallcarca in Barcelona followed the organised tenants of Badalona, thus creating a metropolitan-wide conflict around the same corporate landlord. The collective demand tackled the immediate need of tenants to secure their contractual stability in the face of expiring contracts in the months following the autumn of 2019. They wanted a collective negotiation process with the landlord to establish non-abusive contractual conditions.

Two brief ethnographic descriptions of the days when direct interaction between organised tenants and Azora property managers occurred, on January 2 and February 3, 2022, illustrate how power asymmetry plays into contract-making (see Figure 3). Active contract disobedience of Azora tenants started on January 2, when David and Pablo, two tenants from Badalona, had a meeting scheduled at the local offices of Azora’s real estate managing company, Azzam, part of the same corporate group as Azora. Unlike a regular meeting, the tenants entered the offices accompanied by a group of organised tenants and union members (14 people in total), to the surprise of the office workers. The tenants complained about not having had the time to read the 30-page-long contract beforehand or the opportunity to seek legal advice. They asked if they could bring the contract back home, read it carefully and reschedule the day of the signature. The office boss, who entered the meeting, clarified that the landlords did not authorise them to give the tenants the actual contract and that they could only offer them a template.

Nevertheless, the information on the template was inexact, and the tenants complained. A union member added, “A basic right of consumers is to have access to information before signing a contract. We are asking for something very basic, which is to have the contract in our hands to read it carefully, analyse it, and see what things seem correct and what others don’t.” They concluded, “What we want is to read the conditions of the contract that they are going to sign, not a template.” The office boss insisted, “We put off the signature until Madrid [Azora’s headquarters] says something.”

Separating the formal corporate identity between the landlord company (Azora) and the managing company (Azzam) was a corporate strategy to prevent direct negotiation of the contractual conditions with tenants. Managers’ alleged lack of power to take decisions in regard to contractual conditions worked in practice as a tactic to keep tenants unable to negotiate a slight modification of the lease. Nevertheless, the tenants insisted on the urgency of the matter—“But they are out of contract. It is quite urgent, isn’t it?”—putting into words the anxiety of staying put after the contract had expired, which legally enables the landlord to file a no-fault eviction complaint. Azzam’s office boss finally replied that they could not hand over the original contract because it was presigned by the landlord’s corporate representative. One of the union members reacted with a bold and descriptive claim: “I have a question. A contract is an agreement between two parties, right? Then, if the ‘agreement’ is presigned, as you say—talking to the boss—and if, for instance, on the day of the signature, I want to include changes, how come it is presigned?” The meeting ended, and tenants left the building without signing the contract that, as they felt, landlords were imposing on them.

One month after the first contract disobedience, on February 3, 2020, another tenant, Jose, had a scheduled appointment to sign his new contract. He was also going to disobey the contract’s signature. However, the difference was that the disobedience took the form of an organised action with media presence to increase and rebalance the contract bargaining power in favour of the tenants. A group of more than 50 organised Azora tenants and union activists accompanied Jose to the Azzam office (see Figure 4). The reason for the organised group was that, after sending e-mails and letters seeking collective negotiation of the contract conditions of the tenants, they did not receive a response. Paqui, acting as a spokesperson for Azora tenants, said during a speech:

We have come here today to accompany Jose, because today it was the signature of his new rental contract. A contract that we know is an abusive contract. And we come to say that, behind these abusive contracts, there are pepole, and we are signing contracts that will have an impact on our lives.

The abusive nature of the contract was self-evident, not only in the negotiation process but in the contract’s content. The lawyers of the BTU had detected at least 10 abusive clauses that would later (October 2020) be the object of a collective lawsuit of the tenants against Azora. Among the abusive clauses, we find the compulsory contracting of payment default insurance, the so-called penal clause, which imposes a fine for each day of delay in returning the keys when the contract expires; tenants’ renouncing of their rights in case of supply (water, electricity, gas) interruption; the landlord being afforded the right to go to the tenants’ homes to do periodic revisions without the need of the tenants’ consent; tenants’ renouncing of their right to ask for economic compensation in case of landlord’s contract breach; or a clause stating that all of the clauses included in the contract had been freely negotiated. The last clause ironically expresses the nature of contract domination: it is precisely the intrinsic lack of free negotiation that provoked the imposition of abusive, fraudulent clauses that increased tenant duties while forcing them to renounce their rights and, as a result, confer excess privileges to landlords.

Rental contracts are crucial objects for the constant actualization of contemporary urban enclosures that separate people from their means of subsistence (the home). Enclosures function *via* three acts: the physical-legal act of privatisation, or what we have previously called the historical institution of property rights; the act of dispossession; and finally, the act of subjectivation, which is the “encapturing of people, place, space… within the commodifying and alienating logic of capital accumulation and the competitive, marketizing logic of neoliberal rationality” (Hodkinson 2012, 502). Within subjectivation, contrary to dispossession and separation, we find the element of disciplinary inclusion (Perelman 2000), which, under the appearance of voluntary engagement in a wage or, we could add, a rental relationship, masks the fact that for those who are propertyless, there is no alternative to accessing housing other than signing a rental contract. Contract closures function as actualizations of enclosures. From a landlord’s perspective, the logic behind a contract closure is to find a new, more competitive tenant that will be paying higher rents. For unorganised, isolated tenants under normal market conditions, the constant opening and closing of contracts force them to compete with each other to access housing, having to offer the higher bid rent to stay put.

These competition dynamics become contested by tenants who break with isolation, organise, and protest against it through contract disobedience. As with the case of evictions, the contractual process, when faced with disobedience, is exposed as a form of domination. The unfreedom of the contract is made visible by staying put and refusing the subjective engagement and dispossession (loss of home in case of eviction or income loss in case of rising rents) enacted by the contract enforcement. During the action of the February 3, 2020, the spokesperson of the BTU explained the situation during a speech:

Azora is a vulture fund that has been hoarding housing thanks to the state policies that facilitated it, and that now is extorting families by forcing them to pay rent hikes up to 80% of the rent … We have come today here to let the vulture fund Azora know that it has to respect us. We’ve been weeks, if not months in some cases, simply asking Azora to sit down to negotiate, to negotiate with all the families.” And he added: “”e won’t leave the office until the CEOs of Azora don’t show a minimum will to negotiate. We won’t accept any rent hike or any abusive contractual clause. And if the government does not implement a rent control, if the government does not kick out the vultures, then it will be us, the people, who will do it.

Framing the contractual process as a way of extortion, the tenant movement is not only enacting civil disobedience of contracts but also prefiguring and, at the same time, demanding the legal inclusion of rent controls in order to put restrictions on the content and rules of the contractual process negotiation.

The pressing need for new contracts at fair prices went hand in hand with the demand for a rent control law that, with Azora tenants being the most prominent case of tenant protest and organisation, was finally passed in September 2020 before being cancelled by the Constitutional Court one and a half years later. In the next section, we examine the legal movement to include legal mechanisms that restrict the freedom of contract, of which Azora tenants’ struggle was part.

## Rent control, the legal countermovement, and its detractors

The pursuit of legal reforms of the tenant movement to prevent contractual domination is inserted in a larger, legal countermovement, to use the Polanyian expression, that has attempted to protect—quite literally—habitation (Polanyi 2001) to secure the permanence of people in their homes. As we have seen, this countermovement has a long historical trajectory but finds its most contemporary roots in the current configuration of the housing movement with the creation of the people affected by mortgages (PAH), which back in 2012 started gathering signatures to challenge and change the different laws and regulations that permitted housing precarity, starting with the mortgage law and the eviction rules (D’Adda 2021).

Here we focus on the rules that specifically affect the private rental market and the tenant-landlord contractual relationship. Table 1 reflects the significant legal reforms passed due to the tenant movement’s claims, showing the dynamics of the legal countermovement since the creation of tenants’ unions in 2017. These have mostly revolved around two crucial aspects: on the one hand, to control and lower the price of rents and put an end to the unlimited capacity of landlords to ask for increasingly high rents (A, C, D, E, H, I); and on the other, to restrict tenant turnover by offering longer leases or diminishing the capacity of landlords to evict (vulnerable) tenants (A, B, C, D, G, F, I).

**Table 1.** The legal countermovement: chronology and summary of principal regulations transforming residential rent contractual conditions.^13^

A	October 2018	The Constitutional Court lifts the ban on the Catalan 24/2015 Anti-Eviction Law.	*Llei 24/2015 del 29 de juliol, de mesures urgents per a afrontar l’emergència en l’àmbit de l’habitatge i la pobresa energètica*	Resulting from a popular legislative initiative led by different right-to-housing organisations (PAH, APE), this law, popularly known as the Anti-Eviction Law, forces large landlords (up to 15) to offer social rents (well below market average rents) to vulnerable tenants who are in rental payment arrears or defaults. Beyond rent regulations, the law also includes protective schemes for over-indebted mortgage holders or legal mechanisms to force the social use of vacant housing and bans the interruption of energy supplies of vulnerable people unable to pay the bills.
B	March 2019	Spanish Decree Modifying Urban Urban Rental Law (LAU).	*Real Decreto-ley 7/2019, de 1 de marzo, de medidas urgentes en materia de vivienda y alquiler.*	Extension of minimum duration rental contracts from three to five/seven years. Prohibition of rent increases within the contract years, extension of the minimum notification time before contract termination, and fixing of a maximum deposit claimable in order to access a rental contract.
C	December 2019	Catalan Decree Modifying the 24/2015 Anti-Eviction Law.	*Decret Llei 17/2019, de 23 de desembre, de mesures urgents per millorar l’accés a l’habitatge.*	Contract terminations are being included as situations in which vulnerable tenants can claim a social rent and not just default payment or rent arrear situations.
D	March 2020	Spanish Royal Decree of urgent economic and social measures in the face of Covid-19.	*Real Decreto-ley 11/2020, de 31 de marzo, por el que se adoptan medidas urgentes complementarias en el ámbito social y económico para hacer frente al Covid-19.*	Decree issued by the Spanish Government in order to mitigate the impacts of the social crisis derived from Covid-19. The main rental housing-related measures were rent moratoriums (and, if the landlord was a large landlord, owning 10 or more housing units, tenants could apply for non-mandatory 50% rent release) and the six-months extension of rental agreements that were about to expire.
E	September 2020	Catalan 11/2020 Law, popularly known as Rent Control Law.	*Llei 11/2020, del 18 de setembre, de mesures urgents en matèria de contenció de rendes en els contractes d’arrendament*	Law 11/2020 prohibits rent increases (with some exceptions) and forces rent decreases for rentals that are above the average rental prices marked by the official indicator.
F	November 2020	Catalan Decree which modifies the 24/2015 Social Rent Law, extending the protection of vulnerable tenants against eviction in the context of pandemics.	*Decret Llei 37/2020, de 3 de novembre, de reforç de la protecció del dret a l’habitatge davant els efectes de la pandèmia de la Covid-19.*	Urgent decree issued to increase the protection of vulnerable tenants against evictions by large landlords in the context of the pandemic.
G	December 2020	Spanish Decree of urgent measures to face situations of vulnerability in relation to housing (and transportation), popularly known as the Anti-Eviction Decree.	*Real Decreto-ley 37/2020, de 22 de diciembre, de medidas urgentes para hacer frente a las situaciones de vulnerabilidad social y económica en el ámbito de la vivienda y en materia de transportes.*	Urgent decree issued to increase the protection of vulnerable tenants against evictions by large landlords in the context of the pandemic.
H	December 2020	Catalan Decree modifying the Rent Control Law and urgently stimulating the building of publicly protected rental units.	*Decret Llei 50/2020, de 9 de desembre, de mesures urgents per estimular la promoció d’habitatge amb protecció oficial i de noves modalitats d’allotjament en règim de lloguer.*	Decree that, among other aspects, complements the Rent Control Law (11/2020), making explicit that rents cannot be indirectly increased *via* the inclusion of extra expenses (cleaning service, garbage service, property taxes, etc.).
1	February 2021	The 17/19 Decree, which complements the Social Rent Law 24/2015, is declared unconstitutional by the Constitutional Court. Among other aspects, large landlords do not have the obligation to offer new social rent contracts to tenants when the rental contract expires.		
I	January 2022	Law 1/2022, or the new Anti-Eviction Law.	*Llei 1/2022, del 3 de març, de modificació de la Llei 18/2007, la Llei 24/2015 i la Llei 4/2016 per a afrontar l’emergència en l’àmbit de l’habitatge*	This new Anti-Eviction Law finally consolidates the modifications made by the 17/2019 Decree. Large landlords are obliged to offer a social rent to vulnerable tenants, not only in case of default payment but also after the contract expires.
2	March 2022	The 11/2020 Law or Rent Control Law is declared unconstitutional by the Constitutional Court.		
	May 2023	Passing of the new Spanish Housing Law, which includes a system of rent controls, similar to the 11/2020 Law, prohibiting rent increases (with the exceptions of structural refurbishment) and forcing rent decreases in the case of large landlords (lowered to five or more housing units).		

Source: elaboration from legal texts published in the *boletín oficial del estado*.

The legal countermovement has been met with the stark opposition of pro-market advocates and real estate actors, who pushed for lifting restrictions on the freedom of contract for the sake of the development of the private rental market. In that sense, the numbers in Table 1 (1 and 2) reflect a judicial reaction against the legal transformations, in particular in the form of constitutional appeals (promoted by both the Government of Spain and the main opposition party, *Partido Popular*), against the different regulations. The Constitutional Court has in the last two years (in the 2021–2022 period) ruled against two of the laws, arguably the most ambitious ones: the Anti-Eviction Law (1 vs. C) and the Rent Control Law (2 v.s E). Whereas formal aspects such as the distribution of competencies between state and regional-level legislative capacity were a significant part of the anti-constitutionality of the laws,^7^ the bulk of the public discussion revolves around the more substantive question of the freedom of contract and how these regulations affect private property.

The reaction to the pro-regulation legal countermovement intensified in the context of drafting the first national Housing Law, which was hotly debated in Parliament. The draft, which included a rent control mechanism and measures to prevent evictions of vulnerable families, faced the stark opposition of real estate actors and free-market advocates. The main arguments against the introduction of such measures were twofold: on the one hand, the potential market inefficiencies that the regulation can cause, which boil down to the often-voiced supply reduction due to the fall in returns of rental housing property (Kallin and Slater 2018); and on the other, *inseguridad jurídica* (juridical insecurity) was voiced as a central concern. *Inseguridad jurídica* is a formal expression that technically translates as ‘legal uncertainty’ but whose wording in Spanish, with the emic use of the term ‘security’, made it an even more dramatic claim. Legal certainty is a constitutionally protected principle of the rule of law “which requires that everyone has certainty and knowledge in advance in regard to the legal consequences of his acts and omissions” (Real Academia Española). The way market actors use the expression, nevertheless, slightly drifts away from its formal definition. As reported, Concha Osácar, vice president of Azora, in January 2020, used *inseguridad jurídica* as the cause of investment opportunities being lost, which, in other words, means the reduction of private rental market investors’ profit.^8^ The director of the Association of Developers and Builders,^9^ the president of Alquiler Seguro (managers of private rental units),^10^ and the president and director of the Association of Owners of Rental Units^11^ also made an emic use of the term and claimed that the introduction of rent controls would create a drop in the *seguridad jurídica*, contrary to their investment interests. The Minister of Economy and Finance also stated, “We should avoid evictions, but we should also grant juridical security.”^12^ Pointing to the antagonism between preventing evictions and granting juridical security to landlords, the words of the minister elucidated a crucial dynamic: for real property to function as an asset with stable returns, circulation, and alienability—that is, contract closures and openings *via* property repossession and therefore, evictions—must also be granted.

The debate around drafting the Housing Law (2023) epitomised the legal countermovement and its resistance. The project has been criticised by housing and tenant organisations as insufficient to protect the right to housing and by market actors for its excessive interventionism. The parliamentary debates and the expert commission onthe Housing Law reflected this double criticism. Both experts and members of Parliament who are market advocates pointed to the loss of freedom of contract, the denaturalization, the violation, or even an attack on the right to private property. Market advocates present the protection of private property rights as the optimal and just solution to the housing crisis. Property rights are framed as constitutionally inviolable, while the state administration is solely responsible for providing the right to housing and secure occupancy, which must look after the vulnerable who suffer eviction. Nevertheless, as the analysis has shown, evictions are not accidents essentially related to vulnerable tenants but are a crucial mechanism for contract enforcement and the realisation of contract freedom. Evictions, therefore, cannot meaningfully be framed as a social problem separate from the normal functioning of private property in the rental market.

## Concluding remarks

This article has sought to demonstrate how, on the one hand, the law organises residential rent relations while, at the same time, social relations bring the law to life. Following a critical legal approach, particularly informed by the Marxist legal theory of Pashukanis (1929), we argued that the power of law, as it crystallises in contractual property agreements, is not merely ideological or the direct result of the state monopoly of violence. Law and, in this case, the particular object of the contract and its associated doctrine of freedom of contract are components essential to the unfolding of an economic power (Mau 2021): the contractual mechanism organises social relations in a way that reproduces rent relations, mostly through competition. Contracts, in this light, are legal processes of disciplinary inclusion that, at the same time, allow for continuous exclusion by their opening and closing dynamics, imposing the circulation of tenants and forcing tenant competition to access rental housing under market conditions.

Four conclusions are derived from this point. Firstly, contracts are functional to rent accumulation strategies as they force circulation competition between tenants. The closing and opening of contracts make rent emergent by allowing continuous rent increases to the benefit of the asset holder and by generating and facilitating the expectation of incremental gains from future rent increases. Moreover, this circulation imposed by the contractual logic generates competition between tenants, who are continuously forced to change residences. The rise of tenants’ unions in Spain was a response to this problem by organising tenant resistance in the face of what tenant activists labelled as invisible, no-fault evictions. Secondly, rental contracts are not agreements between equal parties. The autonomy of the will, the assumption that holds the freedom of contract doctrine, does not hold true in the case of tenants. Tenants cannot choose whether they need a house or not. In other words, they are obliged to enter into market relations to provide for themselves this very needed space for their reproduction, the home. From this point, the power imbalance and unequal capacity to bargain the contractual conditions are made evident. As demonstrated in the case of Azora, where tenants protested the clauses that were being imposed on them and sought to negotiate better contractual conditions, the closing and opening of contracts appeared as instances in which the power of the landlord could be exerted. The freedom of contract, in this case, is only freedom for the powerful, real property-owning party, which leaves the tenants bare in front of the pressures of commodification and worsening contractual conditions in general, as exemplified in the case of Azora, with the inclusion of abusive contractual clauses.

The consideration of the power imbalance brings us to the third conclusion, in which the contractual process appears as a mode exerting power that is different and not reducible to either violence or ideology, although both are present in the contractual process. The power of the contract is made evident when evictions take place. Nevertheless, as we have noted, state violence to enforce rental contracts is only exceptionally deployed and must be placed in a wider and longer power-laden contractual process. On the same token, ideology has the capacity to mask the actual, material, and social life of contracts by reproducing ideas about the inviolability of property and the insecurity or uncertainty of legal mechanisms that control the freedom of contract. Nevertheless, such abstractions impregnate political debates and are finally included in legal texts, having a direct impact on social relations and shaping the way contracts are actually implemented. Finally, we reach our last conclusion. The invisibility and pervasiveness of the contractual logic are only broken when tenants associate and mobilise to change the conditions of contract negotiation. Contestation of the freedom of contract has a long history, and tenant movements have shown the capacity to tilt laws in their favour. Nevertheless, freedom of contract remains the default legally codified normality without special regulations such as laws defending tenants’ rights or the right to housing.

## Notes

1 According to the Barcelona Metropolitan Housing Observatory between 2014 and 2018, rents in the private rental market have increased by 33.8% in the metropolitan area of Barcelona. See: https://www.ohb.cat/wp-content/uploads/2019/07/Habitatge_metropolis_2018.pdf. The overburden rate (the percentage of tenant households that dedicate more than 40% of their income to pay rent) has increased from 37.2% in 2018 to 45.6% in 2021.

2 During the fieldwork, I did a close follow-up and gave support to 11 cases (these include either single tenants that have a conflict with their landlord or large tenant communities that are organising against the same (corporate) landlord). The latter is the case that I will use in this paper as an example of tenant mobilisation in the face of contractual negotiations.

3 “To envision housing merely as a “commodity” is, therefore, to lapse into precisely the fetishism that Marx attacked, whereby relations between people – the people who build, own, rent and live in houses – are reduced to the politically and analytically impoverished status of relations between things” (Aalbers and Christophers 2014).

4 It should be nonetheless noted that the work of the legal geographer Nicholas Blomley has stressed the importance of property (Blomley 2005, 2004) as a legal-political arrangement for the defence of placing law, together with political economy, at the centre of analysis of the dynamics of land rent, which in turn is largely absent (Blomley 2020).

5 A 2021 study shows that 45.8% of residential moves are the outcome of an invisible eviction. https://lahidra.net/wp-content/uploads/2021/07/Impactes-socials-del-mercat-de-lloguer-Enquesta-sobre-les-condicions-de-vida-de-la-poblacio%CC%81-llogatera-a-Barcelona-i-la%CC%80rea-metropolitana.-La-Hidra.-.pdf

6 According to official data from the General Council of the Judiciary, in the first term of 2022, 77.47% of the judicial evictions in the residential private rental market resulted from rental payment defaults.

7 Note that the two regulations/laws that the Constitutional Court has ruled out are regional Catalan laws.

8 Expansión; 16/01/2020: “La construcción de pisos cae un 55% en Barcelona y sube un 9% en España” Available at: https://www.expansion.com/economia/2020/01/16/5e1f82b3e5fdeadb228b464a.html [Accessed 2 May 2023].

9 Eje Prime, 10/06/2020: “Gómez-Pintado (Apce) reclama un IVA “superreducido” para el alquiler”. Available at: https://www.ejeprime.com/mercado/gomez-pintado-apce-reclama-un-iva-superreducido-para-el-alquiler.html [Accessed 2 May 2023].

10 El Confindencial, 19/11/2020: “La experiencia europea (y catalana), ¿se constata el fracaso de limitar los alquileres?”. Available at: https://www.elconfidencial.com/vivienda/2020-11-19/alquiler-precios-tensiones-mercado-inmobiliario_2837355/[Accessed 2 May 2023].

11 elEconomista, 26/05/2021: “Asval pide seguridad jurídica para que el mercado del alquiler en España se equipare al resto de Europa”. Available at: https://www.eleconomista.es/empresas-finanzas/noticias/11235852/05/21/Asval-pide-seguridad-juridica-para-que-el-mercado-del-alquiler-en-Espana-se-equipare-al-resto-de-Europa.html [Accessed 2 May 2023].

12 El País, 18/11/2020: “Iglesias presiona al PSOE con los desahucios y Calviño le acusa de crear ‘un conflicto’”. Available at: https://elpais.com/espana/2020-11-18/iglesias-presiona-al-psoe-con-los-desahucios-y-calvino-le-acusa-de-crear-un-conflicto.html [Accessed 2 May 2023].

13 Note that, bearing in mind the need to offer a synthesis, not all of the regulations that have been passed within the analysed period have been included in the chronology, especially regulations that are redundant; that is, decrees that, a few weeks or months after the publication of the main legal text, only seek to improve, complement or extend the applicability of the norms.
